# Going to the Country

**Published:** 1868-06

**Authors:** 


### GOING TO THE COUNTRY.

Good advice has been given in these pages at different times about going to the country during the summer and the best ways of securing the highest amount of enjoyment while we are there, but one " CARL BENSON " makes fun, of us and sends it to the " Evening Post " for every body to read ; we appeal to the public, in justice to ourselves and our known wisdom in such matters, to turn to Health Tract Seventy-nine and compare this Asteroid's article with ours ; and then to accuse us of " plaigarism " its abominable, and then to date it the first of April too! We wish to deal fairly with every body and so give his entire article, verbatim.

